# The effects of a fat loss supplement on resting metabolic rate and hemodynamic variables in healthy females: preliminary results

**DOI:** 10.1186/1550-2783-11-S1-P1

**Published:** 2014-12-01

**Authors:** Gina Zito, Bill Campbell, Ryan Colquhoun, Nic Martinez, Laura Buchanan, Matt Lehn, Mallory Johnson, Courtney St Louis, Yasmin Smith, Brad Cloer, Allison Pingel

**Affiliations:** 1 University of South Florida, Tampa, Florida, USA

## Background

Individuals looking to improve their physique may ingest thermogenic supplements for the purposes of elevating resting metabolic rate and ultimately induce fat loss. The purpose of this study was to examine the effects of a commercially available dietary supplement (containing ingredients that promote thermogenesis) on resting metabolic rate (RMR) and hemodynamic variables in a randomized, double-blind, placebo-controlled cross-over study.

## Methods

10 female participants (27.8 ± 12.4 years; 166.2 ± 7.2 cm; 61.7 ± 8.0 kg, and 22.4 ± 2.8 BMI) volunteered to participate in this investigation. Participants underwent two different testing sessions separated by approximately 7 days. On their first visit, participants arrived to the laboratory after an overnight fast and underwent a baseline RMR, heart rate (HR), and blood pressure (BP) assessment. Following this, each participant ingested a dietary supplement (FitMiss Burn^TM^) or a placebo and repeated the RMR, HR, and BP assessments at 60, 120, and 180 minutes post-ingestion. The thermogenic ingredients contained in the dietary supplement included caffeine, green tea extract, yohimbine HCL, and other ingredients. The placebo was void of active ingredients known to elevate RMR. Approximately 1-week later, the alternative supplement was ingested and the assessments were repeated in the exact same manner. Data were analyzed via a 2-factor [2x4] within-subjects repeated measures analysis of variance (ANOVA) using SPSS version 22.0. Post-hoc tests were analyzed via paired samples t-tests. The criterion for significance was set at p ≤ 0.05. Consent to publish the results was obtained from all participants.

## Results

The repeated measures ANOVA revealed a significance effect for time relative to the raw RMR data. Post-hoc analyses revealed that the dietary supplement demonstrated trends for significance at 60 minutes (p = 0.088) post supplementation and significant elevations in RMR (kilocalories/day) at 2 and 3-hours post ingestion (p = 0.033 and 0.017, respectively) as compared to baseline RMR values. The only elevation in the placebo treatment occurred at 3-hour post supplementation (p =0.024) time point as compared to baseline RMR values. Table 1 demonstrates the raw data (mean ± SD) and the percentage increases in RMR for each time point for both supplement groups. Heart rate and blood pressure values did not change over the course of the 3-hour testing period for either group.

**Table 1:** RMR (mean ± SD kcals/day) and (% increase in RMR as compared to baseline values) for each supplement group

	Baseline	60-minute	120-minute	180-minute
FitMiss Burn^TM^	1,422 ± 221	1,495 ± 182 (5.1%)^#^	1,524 ± 171 (7.2%)^*^	1,526 ± 189 (7.3%)^*^

Placebo	1,425 ± 196	1,464 ± 173 (2.7%)	1,475 ± 173 (3.5%)	1,512 ± 198 (6.1%)^*^

^#^ - Post-hoc statistical trend compared to baseline values (p ≤ 0.10)

^*^ - Post-hoc statistical difference compared to baseline values (p ≤ 0.05)

## Conclusions

The dietary supplement treatment (FitMiss Burn^TM^) experienced greater elevations in RMR values as compared to the placebo treatment. These elevations came with no adverse effects relative to resting heart rate and blood pressure values. Taken on a daily basis, FitMiss Burn^TM^ supplementation may increase overall energy expenditure possibly leading to reductions in fat mass over time.

